# Supplementary material for: Metoprolol Inhibits Developmental Brain Sterol Biosynthesis in Mice
Source: Biomolecules. 2022 Aug 31;12(9):1211. doi: 10.3390/biom12091211 (PMC9496459; doi:10.3390/biom12091211)
Supplement: Supplementary file 1 [file biomolecules-12-01211-s001.zip › biomolecules-1871079-supplementary.pdf]

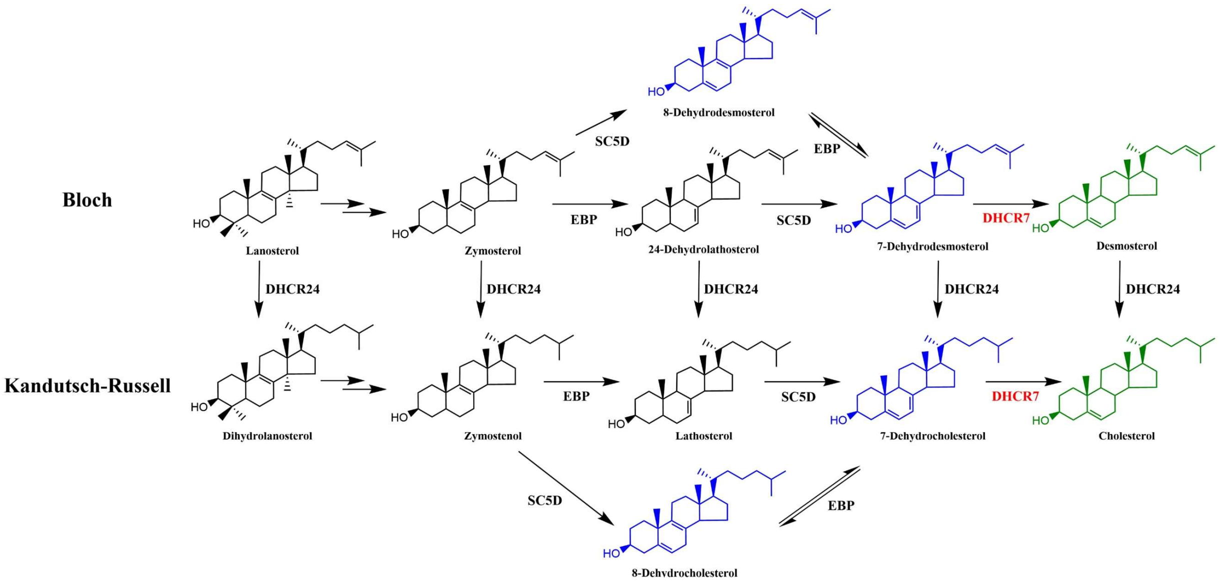

Figure S1. Post-lanosterol cholesterol biosynthesis pathway. Note that DHCR7 (red) is essential for CHOL synthesis though both the Kandutsch-Russell (bottom) and Bloch (top) arms, and that inhibition DHCR7 inhibition result in reduction of product (DES, CHOL; green) and accumulation of precursors (7-DHC, 8-DHC,7-DHD and 8-DHD, blue).

Name	Rank by Rx, 2019	Yearly Rx, 2019	Cross BBB?	Cross placenta?	β-Selectivity	Known CNS side effects
**Metoprolol**	5	74,578,817	Yes	Yes	β1	Dizziness (2% to 10%), fatigue (1% to 10%)
**Carvedilol**	33	20,602,256	Likely yes based off logP	Yes	Non-selective, also α-adrenergic	Dizziness (2% to 32%), fatigue (24%)
**Atenolol**	39	18,091,488	Yes	Yes	β1	Fatigue (≤26%), dizziness (1% to 13%), depression (≤12%)
**Propranolol**	82	9,277,061	Yes	Yes	Non-selective	Sleep disorder (infants: 16% to 18%), Agitation (infants: 5% to 9%), dizziness (4% to 7%), drowsiness (infants: 5%), fatigue (5% to 7%), irritability (infants: 6%), nightmares (infants: 6%)
**Nebivolol**	191	3,061,887	Yes	Yes	β1	Headache (6% to 9%), fatigue (dose-related; 2% to 5%)
**Labetalol**	198	2,825,281	Negligible amounts	Yes	Non-selective	Dizziness (1% to 20%), fatigue (1% to 11%)

Figure S2. Prescription data, action and CNS-related side effects of investigated beta-blockers.

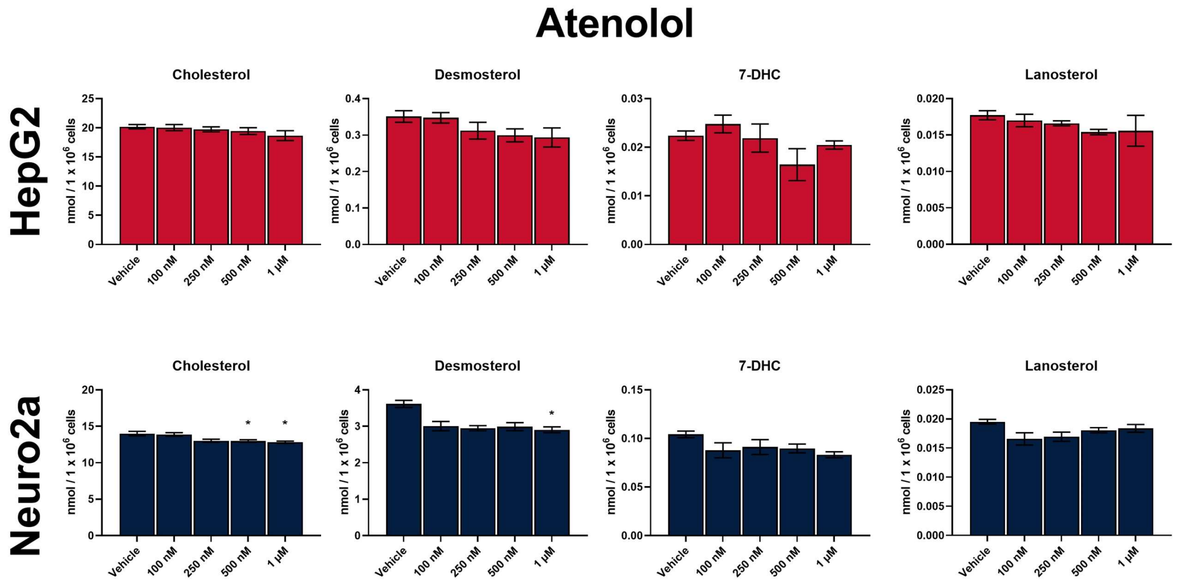

Figure S3. Dose-dependent sterol response of HepG2 and Neuro2a cells treated with atenolol. Values reported as nmol/million cells. Statistical significance: *$p<0.05$; **$p<0.01$; ***$p<0.001$; ****$p<0.0001$ *vs.* vehicle treatment.

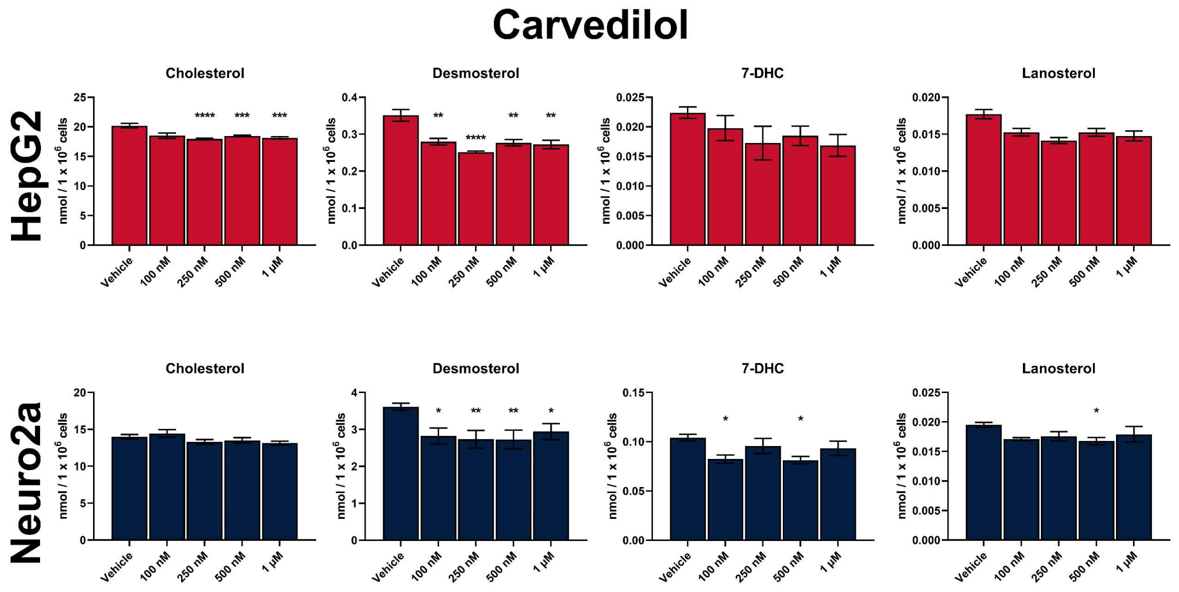

Figure S4. Dose-dependent sterol response of HepG2 and Neuro2a cells treated with carvedilol. Values reported as nmol/million cells. Statistical significance: *$p<0.05$; **$p<0.01$; ***$p<0.001$; ****$p<0.0001$ *vs.* vehicle treatment.

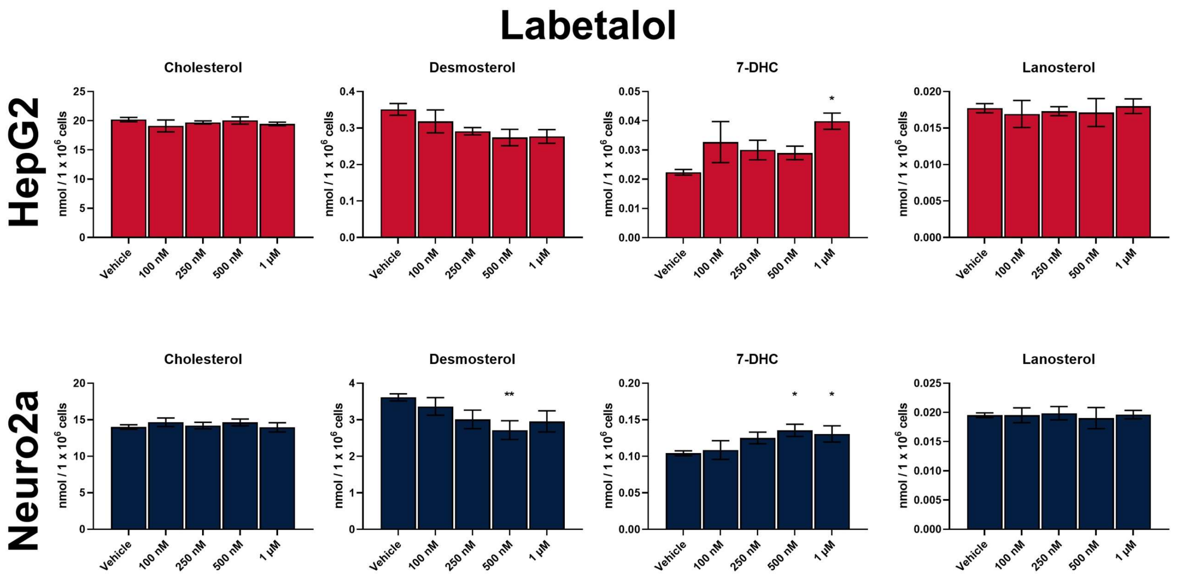

Figure S5. Dose-dependent sterol response of HepG2 and Neuro2a cells treated with labetalol. Values reported as nmol/million cells. Statistical significance: *p<0.05; **p<0.01; ***p<0.001; ****p<0.0001 *vs.* vehicle treatment.

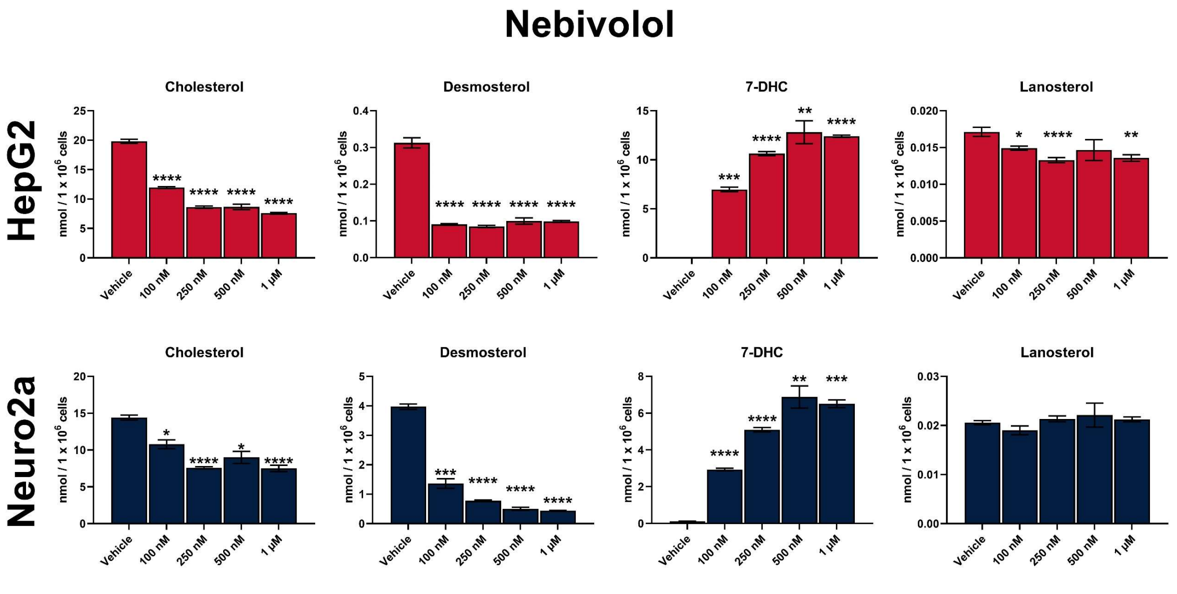

Figure S6. Dose-dependent sterol response of HepG2 and Neuro2a cells treated with nebivolol. Values reported as nmol/million cells. Statistical significance: *p<0.05; **p<0.01; ***p<0.001; ****p<0.0001 *vs.* vehicle treatment.

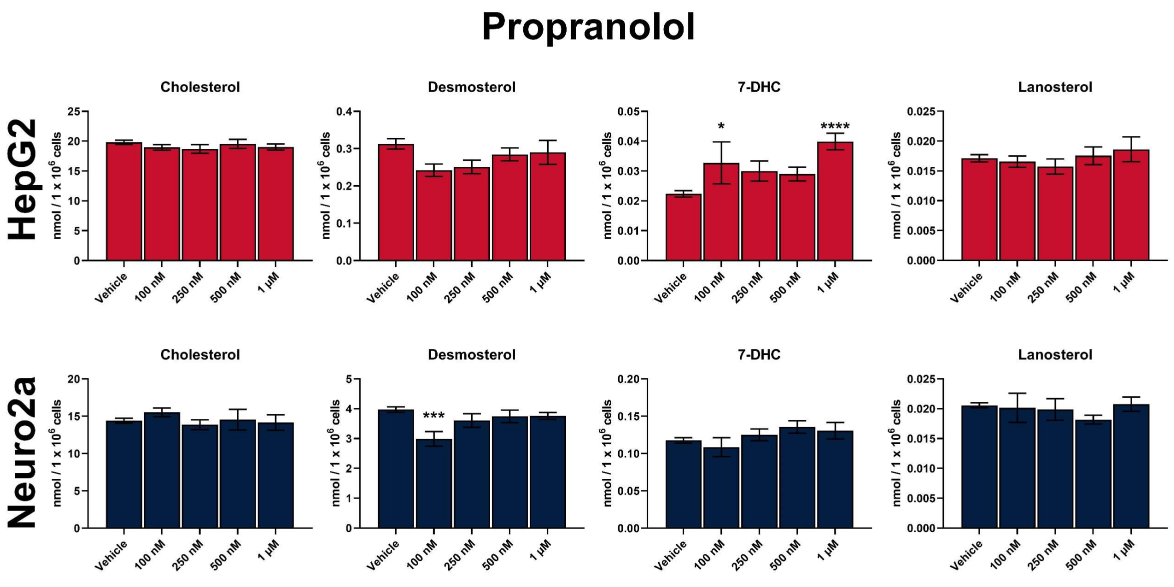

Figure S7. Dose-dependent sterol response of HepG2 and Neuro2a cells treated with propranolol. Values reported as nmol/million cells. Statistical significance: *p<0.05; **p<0.01; ***p<0.001; ****p<0.0001 *vs.* vehicle treatment.

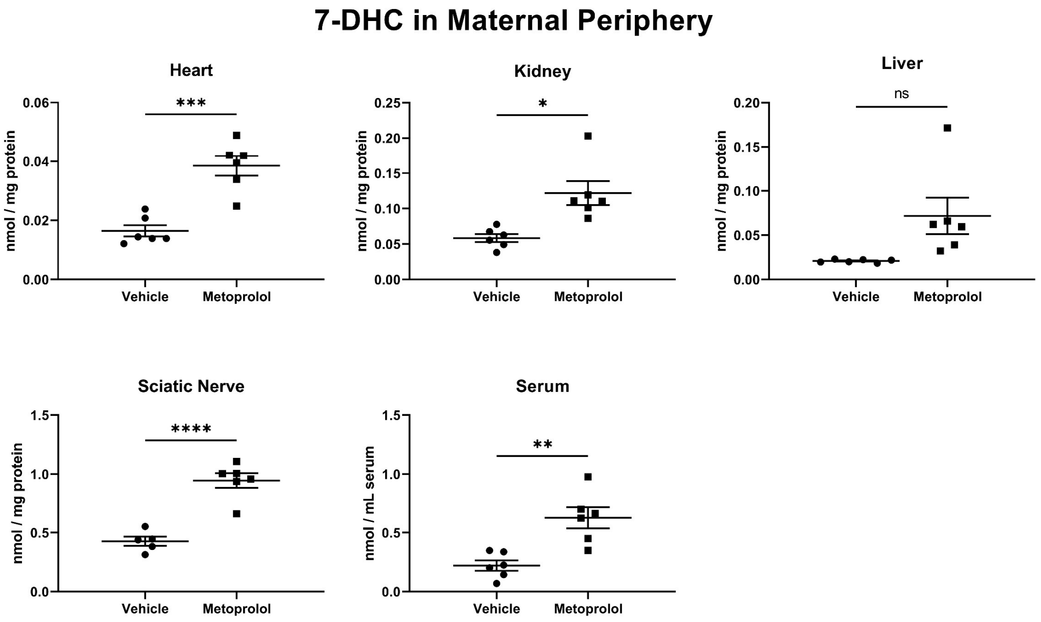

Figure S8. Short-term MTP treatment elevates 7-DHC levels in the peripheral tissues of pregnant mouse dams. Pregnant mice were treated with 35 mg/kg MTP from E12 to E19. Dam tissues were analyzed for sterol content by LC-MS/MS. Statistical significance: **p<0.01; ***p<0.001; ****p<0.0001 vs. vehicle

treatment. **Note** that the 7-DHC elevation is relatively unifrom, and less robust than the elevations seen in the offspring tissues.

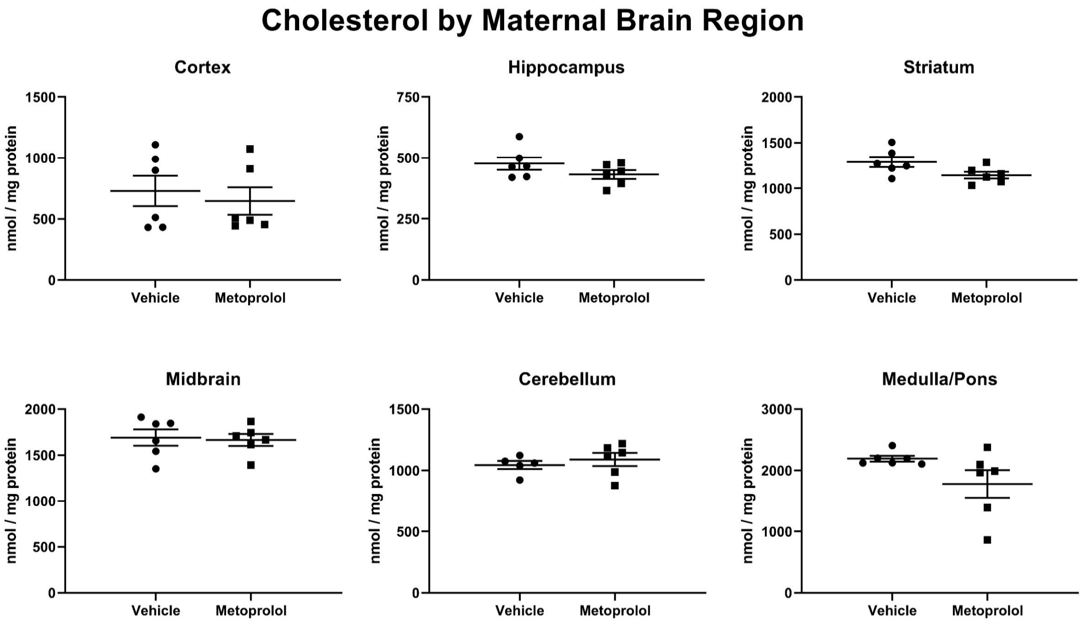

Figure S9. Short-term MTP treatment has no significant effect on cholesterol levels in the brain of pregnant mouse dams. Pregnant mice were treated with 35 mg/kg MTP from E12 to E19. Dam brains were analyzed for sterol content by LC-MS/MS. Statistical significance: **p<0.01; ***p<0.001; ****p<0.0001 vs. vehicle treatment. **Note** that CHOL baseline levels in the vehicle-treated animals are different across the brain regions, and that cholesterol levels are unchanged by short-term MTP treatment.

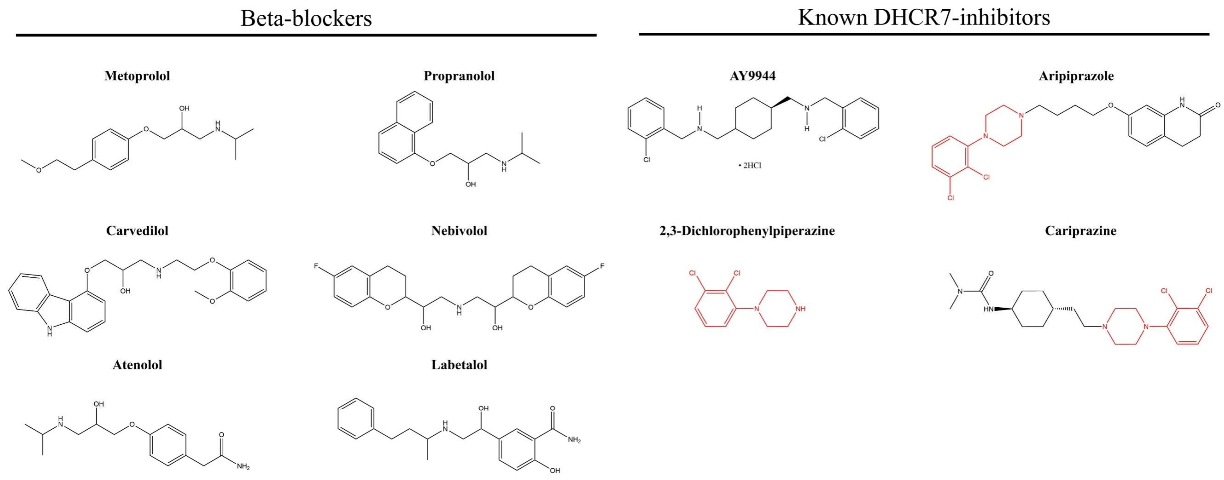

Figure S10. Chemical structures of beta-blockers and known DHCR7 inhibitors. AY9944 is a potent DHCR7 inhibitor commonly used to recapitulate SLOS phenotype in mice. Previous studies have identified aripiprazole, cariprazine and their shared 2,3-dichlorophenylpiperazine moiety (red) as DHCR7 inhibitors. The six beta-blockers we tested do not appear to share this moiety, suggesting that there are multiple classes of chemical structures that might lead to DHCR7 inhibition/ 7-DHC elevation.
